# The Impact of Education Level on Weight Loss in a Primary Care-Anchored eHealth Lifestyle Coaching Program in Denmark: A Randomized Controlled Trial

**DOI:** 10.3390/nu16060795

**Published:** 2024-03-11

**Authors:** Luma Shahin, Thomas Bastholm Olesen, Michael Hecht Olsen, Ditte Hjorth Laursen, Jeanette Reffstrup Christensen, Carl J. Brandt

**Affiliations:** 1 Research Unit for General Practice, Department of Public Health, University of Southern Denmark, 5230 Odense, Denmark; jrchristensen@health.sdu.dk (J.R.C.); cbrandt@health.sdu.dk (C.J.B.); 2 Department of Internal Medicine, Holbaek Hospital and Steno Diabetes Center Zealand, 2730 Herlev, Denmark; michael.olsen@dadlnet.dk; 3 Steno Diabetes Center Odense, Odense University Hospital, 5000 Odense, Denmark; thomas.bastholm.olesen@rsyd.dk; 4 Department of Clinical Medicine, University of Copenhagen, 2200 Copenhagen, Denmark; 5 Liva Healthcare, Research and Innovation, 1434 Copenhagen, Denmark; dl@livahealth.com; 6 DRIVEN, Institute of Sports Science and Clinical Biomechanics, University of Southern Denmark, 5230 Odense, Denmark; 7 Research Unit of General Practice, 8000 Aarhus, Denmark

**Keywords:** digital behavioral coaching, health behavioral change, obesity, type 2 diabetes

## Abstract

In a randomized controlled trial including 340 people living with obesity, with and without type 2 diabetes, digital coaching has induced significant long-term weight loss compared to the usual methods of care. We investigated whether education level influenced this weight loss and which lifestyle changes supported the digital lifestyle coaching program. The intervention consisted of a 1 h face-to-face motivational interview followed by digital coaching using behavioral change techniques. At 6 months, the weight loss in the intervention group was significantly larger in participants with short education (6.0 vs. 2.2 kg, *p* < 0.01) (*p* = 0.006). Participants with long education experienced initially a modest weight loss, but the effect was maintained, leading to the largest weight loss at 24 months (5.06 [−11.98–1.86] kg), even though there were fewer coaching sessions in the maintenance period. In multiple regression analyses, the greater weight loss in the intervention group was associated with short education (*β* = 1.81, *p* = 0.02), improvements in everyday physical activity (*β* = 2.60, *p* = 0.014) and improvements in dietary habits (*β* = 3.84, *p* = 0.013). In conclusion, at 6 months, the effect of the intervention was more pronounced in people with short education through improvements in everyday physical activity and dietary habits. However, participants with long education sustained their weight loss at 24 months.

## 1. Introduction

The global incidence and prevalence of type 2 diabetes (T2D) has increased significantly over the last several decades [1]. The total population of adult patients with diabetes in Denmark is estimated to be around 350,000 (6.2% of the entire population), and approximately 89% of these have T2D [2]. More than 80% of all patients with T2D are overweight or experiencing obesity [3]. Obesity and inactivity are of major importance for developing T2D [4]. Self-management, including healthy lifestyle, is thus essential for prevention and treatment of T2D [3].

Despite an intensive focus on T2D in general practice, treatment goals have not been achieved for many patients in Denmark [5]. Studies have shown that annual consultations seldom address lifestyle issues, nor do the patients follow recommendations for a healthy lifestyle, such as physical activity recommendations or tobacco cessation [5,6]. Systematic reviews and meta-analyses show that electronic health (eHealth) solutions are significantly better than usual care, defined as routine diabetes self-care in general practice with no personalized feedback when supporting weight loss short term (3–6 months) in participants with obesity (BMI ≥ 30) [7,8,9]. Previously, in a randomized controlled trial (RCT) called ‘long-term Lifestyle change Intervention and eHealth Application’ (LIVA), we have shown that digital lifestyle coaching can induce and maintain significant weight loss compared to usual care for 12 months (4.5 [3.4–5.6] kg vs. 1.5 [0.2–2.7] kg) in people living with obesity, both with and without T2D [10].

However, the effect of using digital coaching, like any other intervention, is dependent on the participants’ adherence to the intervention, emphasizing the importance of participant motivation and coping resources, which often are related to socioeconomic and psychological factors [11]. Moreover, good quality evidence-based interventions are lacking in individuals experiencing obesity with a lower socioeconomic status. One study has demonstrated that eHealth weight loss interventions can lead to a short-term increase in physical exercise and weight loss in people with a low socioeconomic status (SES) [12], but also that more thoroughly designed studies of longer duration are needed [12]. We hypothesize that similar weight loss will be observed across various education levels in participants using the eHealth application (LIVA), as digital coaching seems to be also effective in participants with a low socioeconomic status.

Therefore, with the present pre-specified post hoc analysis, we aimed firstly to investigate at which time-points the digital lifestyle coaching program LIVA was most effective in reducing weight in various education level groups in obese participants compared to usual care. Secondly, we investigated which lifestyle changes were best supported by the digital lifestyle coaching program.

## 2. Materials and Methods

### 2.1. Study Design and Ethical Approval

The LIVA study was an RCT carried out from March 2018 to October 2021 in the Capital Region of Denmark and the Region of Southern Denmark. The two regions include a total of 40 municipalities. The study was approved by the scientific committee of the Region of Southern Denmark (S-20170183G) and registered in clinicaltrials.gov (NCT03788915). All methods are described in detail in the study protocol [13] but will be presented here in short.

### 2.2. Participants and Eligibility Criteria

**Figure 1:**
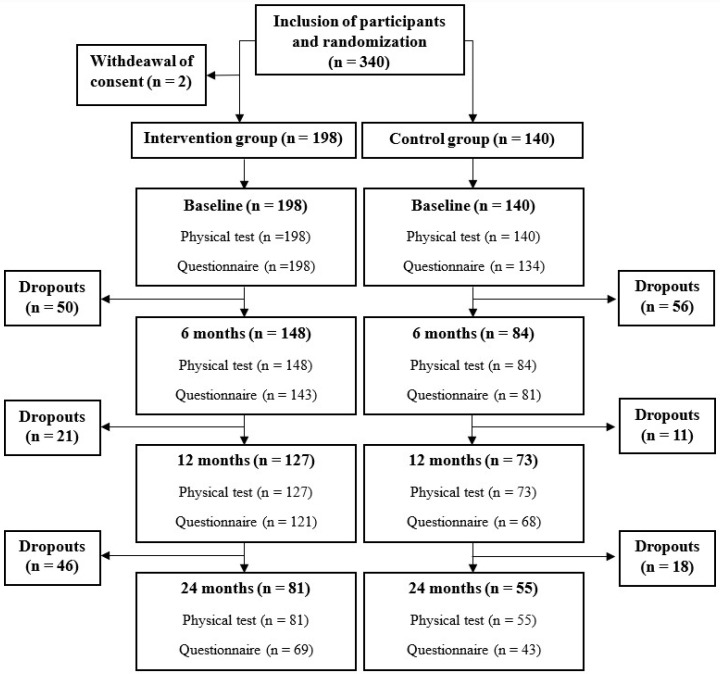
Flowchart of participation and questionnaire completion from baseline to 24 months in the randomized controlled trial.

All participants were recruited either through their general practitioners (GPs), their local health center (there is one in each municipality), the Danish Diabetes Association, or advertisements via social media. Participants who were interested in participating could then register through the LIVA Healthcare app. After registration, participants were contacted by telephone by a research assistant, who ensured that the participants met the inclusion criteria of (a) a body mass index (BMI) of 30–45 kg/m^2^ and (b) an age between 18 and 70 years. The following exclusion criteria were applied: (a) lack of internet access through a computer or smartphone, (b) pregnancy or planned pregnancy, or (c) serious or life-threatening disease defined as a less-than-one-year lifetime expectancy. A total of 340 participants were included. Two participants in the intervention group decided to withdraw their consent; thus, a total of 338 participants were randomized. At baseline, the intervention group included 198 participants and the control group included 140 participants (Figure 1).

### 2.3. Randomization

Participants randomized to the intervention group received digital lifestyle coaching in addition to usual care, whereas participants randomized to the control group received only usual care. Randomization occurred after the participants had completed a medical examination via an automated computer algorithm in groups of 10 at a 6:4 ratio, where 60% of the recruited participants were randomized to the intervention group and the remaining 40% were assigned to the control group. The allocation method was based on a pilot RCT [14] and is described in detail in our protocol paper [13]. Randomization was controlled to ensure that 50% of participants in both the intervention and control group previously had been diagnosed with T2D. After randomization, blinding for the intervention was not possible, but neither the research assistant who carried out the tests nor the intervention health coach had a role in analyzing or interpreting the data [13].

### 2.4. Outcomes and Data Collection

All participants gave their written consent and informed the research assistant about their medication use at the baseline meeting, where the physical examination was performed. The examination included such measurements as the participants’ height, measured in centimeters without shoes, and weight, measured with clothes but without shoes while subtracting 1 kg for clothing. Height and weight were used to calculate the Body Mass Index (BMI = kg/m^2^) [9]. Additional examinations were made but not described in the present study since they were not relevant to our two aims; however, they are described in our protocol paper [13]. The examination was also performed after 6, 12 and 24 months [13].

The participants then filled out an online questionnaire assessing a range of measures included in our protocol paper [13]. The data relevant to the two aims in this paper includes sociodemographic data, such as education level, marital and employment status, and lifestyle habits such as physical and dietary behaviors [Table S1]. The dietary questions are based on the models established by the Swedish National Board of Health and Welfare (SoS) [15].

Each question had several answer options. However, after receiving the answers from the participants, some answers were combined to create a more logical and clear division of categories. In marital status, the three answers ‘Single’, ‘Widow or longest living partner’, and ‘Divorce or no longer in registered partnership’ were combined to create the category ‘Single, divorced or widow’. In employment status, the two answers ‘On the labor market’ and ‘Student’ were combined to create a category named ‘Employed or student’, whereas the two answers ‘Unemployed’ and ‘Retired’ were combined to create the category, ‘Unemployed or retired’. Questions about dietary habits, such as how often participants eat vegetables (fresh as well as frozen) and how often they eat fruit (fresh, frozen, preserves, or juice/smoothie), had four answer options, while the two answer options ‘Twice daily or more often’ and ‘Once a day’ were combined to create the category ‘Once a day or more often’. The question ‘How often do you eat seafood as a main course?’ had four response options, and ‘Twice a week’ and ‘Once a week’ were combined to create ‘Once or twice a week’. The question ‘How often do you eat pastry, chocolate, sweets and/or drink soda?’ had four response options, and the two ‘Daily’ and ‘Almost every day’ were combined to create the category ‘Daily or almost every day’.

The ‘0 min/no time’, ‘Less than 30 min’, ‘30–60 min (½–1 h)’, ‘60–120 min (1–2 h)’, and ‘More than 120 min (2 h or more)’ answers about physical activity were combined to create the three categories ‘Less than 30 min’, ‘½–2 h’ and ‘2 h or more’, while ‘0 min/no time’, ‘Less than 30 min’, ‘30–60 min (½–1 h)’, ‘60–90 min (1–1½ h)’, ‘90–150 min (1½–2½ h)’, ‘150–300 min (2½–5 h)’, and ‘More than 300 min (5 h or more)’ were the answers about everyday physical activity that were also combined to create the three categories ‘Less than 30 min’, ‘½–2½ h’ and ‘2½ h or more’.

The participants answered the questionnaires at each of the four clinical examinations. The participants were contacted one month before their 6-, 12- and 24 month assessments by telephone to schedule the assessment. If a participant did not respond, a voice mail was left explaining the purpose of the call. Another telephone call was made a week later, then again after another week after that, and then again one month later. Participants who had not responded to the four different attempts were considered lost to follow-up.

### 2.5. Intervention Group

After downloading the LIVA app, the participants in the intervention group received an initial 45- to 60-min lifestyle-coaching consultation with the health coach online or in person. Based on the participants’ individual wishes, goals were created using the SMART (specific, measurable, attainable, relevant, timely) model [16].

In the initial consultation, a partnership was created between the coach and the individual in question, guiding them through the Stages of Change (SoC) [17], developing a lifestyle plan around personalized user-centered goals that can be tracked over time, together with educational resources specifically tailored to the needs of the participant. The goal of the health coach was to inspire the participants, commend on goal attainment, and help the participants to stay motivated [18]. The suggestions for new lifestyle behavior were based on a comprehensive theoretical model developed specifically for the LIVA coaching program that combined a range of theories such as the Social Cognitive Theory [19], Self-Determination Theory (SDT) [20], a Patient Centered Communication (PCC) approach [21], the BCT taxonomy [22], and Motivational Interviewing (MI) [23]. The program was delivered by trained health coaches who had a healthcare professional background. Based on the goals and needs of the participant, the coaches drove the user’s engagement using the theoretical foundation in combination with goal tracking, personalized content, and progress in the program. The subsequent asynchronous eHealth coaching sessions were carried out once a week for the first 6 months, then once a month for the next 6 months. Finally, as maintenance, participants only received coaching every third month from 12–24 months.

### 2.6. Control Group

All participants randomized in the control group were invited to follow-up examinations at the same frequency as the intervention group, and they also answered the questionnaires at each timepoint. The participants in the control group were not offered access to the LIVA app but received only the usual care preferred by the patient and their doctor.

### 2.7. Characteristics of Health Coaches

The health coaches who provided the digital lifestyle coaching through the LIVA app were all educated as either nurses, physiotherapists, dieticians, or occupational therapists. They all had received special training on digital health coaching and had practiced that for at least two years. Each patient had a primary health coach, creating the opportunity to form a close and trusting relationship [13].

### 2.8. Statistical Analysis

All analyses were performed using Stata version 18.0 (StataCorp LLC, College Station, TX, USA). Baseline characteristics were presented as means with standard deviations (SD) for continuous variables and as percentages for non-continuous variables. Unpaired Student-*t* tests or Chi^2^-tests were used to compare participants attending or not attending their 6-, 12-, and 24 month examination, respectively.

Reduction in weight was calculated as weight at baseline minus follow up at 6, 12, and 24 months and presented in kilograms with 95% confidence intervals. Unpaired Student-*t* tests or Chi^2^-tests were used to compare change in weight in the intervention and the control group. Interaction analysis was used to compare weight loss in different subgroups. Statistical significance was set at 2-tailed *p* < 0.05.

Chi^2^-tests were used to compare the fraction of participants reporting improvements in physical and dietary behaviors in the intervention and control group, and these results were presented as percentages.

Finally, univariate and multivariate regression analyses were used to determine the association between weight loss as the dependent variable and baseline characteristics and changes in lifestyle at 6, 12, and 24 months of follow-up as the independent variables.

### 2.9. Creating New Variables

The original answers on physical exercise, everyday physical activity, and dietary habits from baseline to the 6-, 12- and 24 month follow-up examinations were changed to a semi-continuous variable by giving each answer a value from 1–5, 1–7 or 1–4, where the best answer had the highest value. Changes were calculated by subtracting baseline values from values obtained from the 6-, 12-, and 24 month follow-up examinations. These changes were then divided into three categories (improved (value > 1), stable (value = 0), worsen (value < 0) and used to compare the fraction of participants reporting improvements in physical and dietary behaviors, helping to determine the association between weight loss and changes in lifestyle. However, participants reporting the highest or lowest values at baseline could not experience improvement or worsening, respectively. Therefore, we performed a sensitivity analysis defining a continuing highest or lowest value as improved or worsened, respectively.

Moreover, all four questions about dietary habits (Table S1, question 4–7) were combined to create an overall diet variable. The new variable was divided in two categories: healthy (value > mean value) and unhealthy (value < mean value).

## 3. Results

### 3.1. Participants Characteristics

Participants who did not participate in the 6-, 12-, or 24 month follow-up examinations were generally not different from the attending participants (Table 1). However, a larger fraction of the participants coming to the 6- and 12-month follow-ups were married or in a registered partnership. There was no difference in education level or other socioeconomic characteristics between the 136 (40.2%) participants attending and the 202 (59.8%) participants not attending the 24 month follow-up examinations (Table 1).

### 3.2. Measured Weight Loss in Different Subgroups

**Figure 2:**
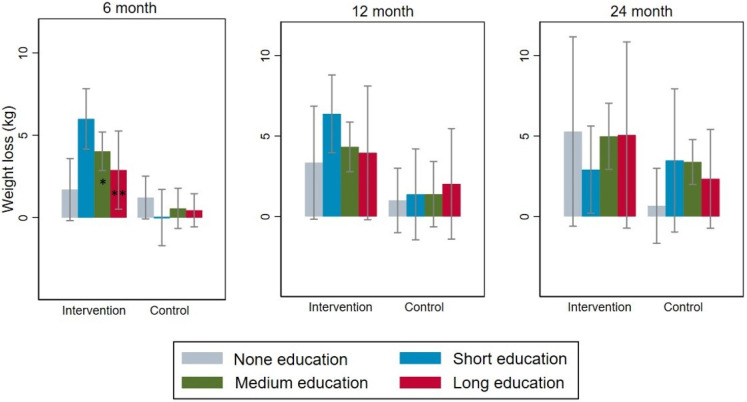
Weight Loss According to Education in Intervention and Control Groups at the 6-, 12-, and 24 Month Follow-Up Examinations. * *p* = 0.006 for the interaction between short education and weight loss in the intervention group vs. control group at 6 months. ** *p* = 0.10 for the interaction between medium education and weight loss in the intervention group vs. control group at 6 months.

In general, the greater weight losses within the intervention group at 6 and 12 months were independent of baseline characteristics (Table 2). However, at 6 months, the weight loss in the intervention group was significantly larger in participants with short education (5.99 vs. 2.20 kg, *p* < 0.01) (*p* = 0.006 for interaction) (Table 2) (Figure 2). At 6 months, there was also a tendency towards smaller weight loss in participants undertaking moderate physical exercise at baseline (3.24 vs. 1.62 kg, NS) compared to participants with low physical exercise (4.20 vs. 0.05 kg, *p* < 0.01, *p* = 0.08 for interaction) (Table 2). At 6 and 12 months, the weight loss in the intervention group was significantly larger both in participants with type 2 diabetes (T2D) (4.00 vs. 1.63 kg, *p* < 0.01 and 3.98 vs. −0.29 kg, *p* < 0.01) or without T2D (4.50 vs. 2.09 kg, *p* = 0.46 and 4.64 vs. 0.68, *p* < 0.01) compared to the control group without any interaction (Table 2). Moreover, at 24 months, weight was more reduced in participants younger than 54 years (5.54 vs. 1.46 kg, *p* = 0.03) compared to participants older than 54 years (3.21 vs. 3.66 kg, NS) (*p* = 0.052 for interaction) and in single participants (including divorced and widowed) (5.80 vs. 0.28 kg, *p* < 0.01) compared to married participants (including registered partnership) (3.77 vs. 3.47 kg, NS) (*p* = 0.04 for interaction) (Table 2).

### 3.3. Self-Reported Improvement in Activity Levels and Dietary Habits

At the 6 month follow-up examinations, the intervention group compared to the control group improved everyday physical activity, and dietary habits by eating more vegetables and/or root vegetables, and significantly more in participants with short (50.0 vs. 22.7%, *p* = 0.04 and 50.0 vs. 22.7%, *p* = 0.04) or medium-long education (46.0 vs. 28.6%, *p* = 0.07 and 48.7 vs. 23.8%, *p* = 0.01) (Table 3).

In addition, more participants with medium-long education also improved their dietary habits by eating more fruits and/or berries (37.8 vs. 21.4%, *p* = 0.07) or less pastry, chocolate, sweets and/or drinking soda (52.7 vs. 26.2%, *p* = 0.01) (Table 3). At 12 months, the intervention still improved everyday physical activity in significantly more participants with medium-long education (51.7 vs. 29.0%, *p* = 0.04). Additionally, at 12 months the intervention was associated with improving physical exercise in more participants with long education (69.2 vs. 22.2%, *p* = 0.03) (Table 3).

### 3.4. Correlation between Weight Loss, Baseline Characteristics and Behavioral Changes

For the whole population, the weight loss at 6 months was larger in participants with lower BMI (*β* = 0.18, *p* = 0.04) or of older age (*β* = 0.07, *p* = 0.03) at baseline, as well as in participants with steady or increasing everyday physical activity (*β* = 1.93, *p* = 0.025; *β* = 2.35, *p* = 0.003) and who were eating more vegetables (*β* = 3.20, *p* = 0.004) or fruits (*β* = 2.08, *p* = 0.039) or eating less pastry, chocolate, sweets or drinking less soda (*β* = 2.31, *p* = 0.035) (Table 4). In the multiple regression analyses, the weight loss in all participants was mainly associated with steady or increasing everyday physical activity (*β* = 1.57, *p* = 0.05; *β* = 1.68, *p* = 0.022), eating more vegetables (*β* = 2.46, *p* = 0.018), or receiving the intervention (*β* = 2.94, *p* < 0.01) (Table S2).

At 6 months, achieving a larger weight loss in the intervention group was associated with improvement in everyday physical activity (*β* = 2.77, *p* = 0.01), as well as eating more vegetables (*β* = 4.09, *p* = 0.010) and not eating more pastry, chocolate, sweets and/or drinking soda (*β* = 2.81, *p* = 0.04; *β* = 3.68, *p* = 0.007). Moreover, there was a tendency towards greater weight loss by having a short education level (*β* = 1.96, *p* = 0.06) (Table 4). In the multiple regression analyses, the weight loss in the intervention group was mainly associated with short education level (*β* = 1.81, *p* = 0.02), improvement in everyday physical activity (*β* = 2.60, *p* = 0.01), and eating more vegetables (*β* = 3.84, *p* = 0.01) (Table S2). In the control group, baseline characteristics such as lower BMI at baseline (*β* = 0.20, *p* = 0.048), being unemployed or retired (*β* = 1.72, *p* = 0.03) and having type 2 diabetes (*β* = −1.92, *p* < 0.01) was associated with greater weight loss at 6 months (Table 4).

For the whole population, the weight loss at 12 months in the multiple regression analysis was mainly associated with steady (*β* = 2.66, *p* = 0.024) or increasing everyday physical activity (*β* = 2.49, *p* = 0.016), eating less pastry, chocolate, sweets or drinking less soda (*β* = 4.82, *p* = 0.003), or receiving the intervention (*β* = 2.75, *p* = 0.003) (Table S3). At 12 months, achieving larger weight loss in the intervention group was negatively associated with steady levels of physical activity (*β* = −0.70, *p* = 0.025) (Table S4). However, this became non-significant in the multiple regression analyses. In the control group, eating less pastry, chocolate, sweets or drinking less soda (*β* = 5.70, *p* = 0.006) was associated with greater weight loss, also in the multiple regression analysis (Table S4).

At 24 months, not eating more pastry, chocolate, sweets or drinking less soda was associated with larger weight loss for the whole population (*β* = 4.95, *p* = 0.02; *β* = 4.56, *p* = 0.04) and in the intervention group (*β* = 5.61, *p* = 0.055; *β* = 6.06, *p* = 0.059). For the control group, a larger weight loss was associated with older age (*β* = 0.13, *p* = 0.029) (Table S5).

### 3.5. Sensitivity Analyses for the Correlation between Weight Loss and Behavioral Changes

At 6 months there was a shift in the significance of some of the associations in Table 4. The association between weight loss and improved vs. worsened physical exercise for the entire population was initially not statistically significant (*β* = 1.70, *p* = 0.09), but it became significant (*β* = 1.66, *p* = 0.04). Similarly, stable vs. worsened levels of everyday physical activity was initially significant (*β* = 1.93, *p* = 0.03), but became non-significant (*β* = 1.67, *p* = 0.056). In the intervention group, stable vs. worsened levels of physical exercise changed from being significant (*β* = 2.70, *p* = 0.05) to becoming non-significant (*β* = 2.10, *p* = 0.11). Additionally, the consumption of more vs. less seafood, both for the overall population and the control group, shifted from being non-significant (*β* = −1.15, *p* = 1.15 and *β* = 0.04, *p* = 0.98) to becoming significant (*β* = 1.67, *p* = 0.04 and *β* = 1.93, *p* = 0.04). Minor changes were also observed by performing sensitivity analyses for the associations between weight loss and behavioral changes at 12 months.

## 4. Discussion

### 4.1. Principal Findings

The weight loss of the intervention group after 6 months was significantly larger in participants with a short education compared to other education levels. This initial weight loss was mainly associated with improvement in everyday physical activity and dietary habits, such as eating more vegetables and/or root vegetables and more fruits and/or berries, which was more pronounced in participants with short or medium-long education. However, this initial larger effect of the intervention group in participants with a short education compared to other education levels was, despite a significant weight loss at 12 months, no longer significant at the 24 month follow-up examinations.

### 4.2. Comparison with Prior Research

Our findings are new, as Myers-Ingram et al. concluded in their systematic review from 2023 that it was unknown whether any eHealth interventions could facilitate weight loss and physical activity in people with obesity with low SES [12]. The LIVA Healthcare app is a hybrid model based on the relationship between patients and coaches, which might explain why it, over a 12 month period, supported individuals with short education and potentially low SES. It may also have supported individuals long-term if the intervention was sustained [8].

Moreover, individuals with a short education and/or low SES often have little flexibility in their time-schedule for work, making it difficult to attend to routine diabetes self-care sessions [24]. Therefore, eHealth solutions can be one approach to overcome these barriers faced by individuals with a short education and/or low SES by allowing them to access weight management interventions at any time and location.

Our results showed that individuals with a short education and probably lower SES improved everyday physical activity and dietary habits by using the LIVA app compared to controls. This may be because the LIVA Healthcare app deploys small changes when supporting everyday physical activities, such as a walking, light gardening, cleaning, or cycling to and from work [18]. Similar findings were concluded in a 24 month RCT from 2012 [25]. Bennett CG et al. evaluated the effectiveness of an eHealth weight loss intervention in 365 participants with obesity, whereas 86.3% had an education level lower than a college degree. They found a larger weight loss at 24 months in the intervention group compared to the control group (difference, −1.03 [−2.03 to −0.03] kg) [25]. Thus, people with a shorter education and probably lower SES have generally less flexible working hours, which can lead to usual care failing in supporting lifestyle changes and weight loss. A wide-reaching eHealth solution may therefore be the answer for participants with short education and lower SES. However, to maintain the effect, coaching must continue, as the effect diminishes if unsupported.

Participants with long education experienced a modest weight loss by the intervention in the first year, but the effect was maintained, leading to the greatest weight loss after 2 years (5.06 [−11.98–1.86] kg). Moreover, they were the only participants who showed improvement in physical exercise after 1 year, even though there were fewer coaching sessions. This suggests that participants with long education had a more steady but sustainable weight loss, and this appears to be associated with improvement in physical exercise of a certain intensity.

It is not unusual for participants with longer education and/or high SES to maintain weight loss independently of the method used. Serdula et al. reported that the odds of trying to lose weight vs. doing nothing about the weight increased with education level [26]. Moreover, using physical activity and exercise as a strategy to lose weight also increased with education level [26].

Earnest et al., in a retrospective analysis in 2020, examined the effects of online weight loss programs by accounting for class attendance and education level, concluding that online weight loss programs are effective regardless of education level [27]. However, class participation was essential to participant success [27]. The study suggested that individuals from higher SES compared to lower SES have lower dropout rates in health promotion programs. Higher attendance and adherence to weight loss programs may be the reason why people from higher SES experience better long-term effects, indicating that extended intensive coaching may not be necessary for this group [12]. In our study, the dropout rate was independent of education. However, we saw an insignificantly higher initial dropout rate in participants with long education, whereas participants with short education had a higher dropout rate after one year, suggesting that extended coaching is more important for participants with short education.

### 4.3. Strengths and Limitations

The dropout rate at 24 months was 202 of 338 (59.8%), which is high but not unlike the adherence rates reported in other studies [8]. Although this could have created adherence bias, the participants who came to the 24 month follow-up examinations were not different in baseline characteristics from those who dropped out. This random dropout supports that the high dropout rate may primarily have occurred due to the COVID-19 lockdown and national restrictions. In hindsight, we should have gathered information about this dropout, but did not due to the focus being on completing the study despite the lock down. This relatively high number of dropouts reduced the power by making the education subgroups small, increasing the risk of type 2 errors with a higher risk of false negative results, especially in years one and two.

A limitation of this study is the way participants reported their improvements in physical activity and dietary habits in the questionnaires. Participants are required to categorize their activity levels within certain time intervals rather than reporting how much time they spend per week on physical activity in minutes. If a participant improves in physical activity by spending more than 2 h per week on physical exercise, they cannot report this change, and, similarly, if they decrease in physical activity by spending less than 30 min per week on physical exercise, they cannot report that change either. This may result in not detecting those participants who improved or worsened from these outlier points. Moreover, the changes in physical and dietary behaviors between baseline and the 6-, 12- and 24 month follow-ups were based on simple subtraction, leading to three categories (improved, stable, or worsened) that did not distinguish between small and large changes, nor between stable values among the lowest (could not worsen) or highest values (could not improve). This may have reduced the number of participants experiencing an improvement, which may have reduced our statistical power. Therefore, we conducted a new analysis where participants with continuing highest or lowest values were defined as improved or worsened, respectively. This new analysis did not lead to major changes in the overall results; however, for the entire population and the control group, improvements in dietary habits such as consuming more seafood were associated with greater weight loss at both the 6- and 12 month follow-up examinations. This association was not observed in the intervention group, suggesting that the weight loss in the intervention group was primarily achieved through increased levels of everyday physical activity.

Lastly, although the effect of education on weight loss was analyzed, other socioeconomic aspects were not assessed, as none had been collected, unfortunately. This could be a limitation, as other socioeconomic aspects may have influenced the effect of the intervention on participants.

The strengths of this study were the very high answering rate of questionnaires about lifestyle habits and sociodemographic aspects at 6 months (96.6% in the intervention group vs. 96.4% in the control group), 12 months (95.2% vs. 93.2%), and 24 months (85.2% vs. 78.2%). At baseline, we have questionnaires for all participants, as this was a requirement for participation. However, six participants in the control group did not report their education level.

Lastly, the long duration of the follow-up made it possible to determine the long-term effect of an eHealth intervention on weight loss and lifestyle habits in people with different SES, which is seldom achieved [12].

## 5. Conclusions

Evidentially, the digital lifestyle coaching program LIVA pronouncedly supported weight loss for individuals with short education at 6 months and 12 months, correlating this success with increased physical activity and dietary improvements at 6 months. Nevertheless, those with higher education persisted in losing weight over 24 months, indicating the intervention’s flexible efficacy across different educational levels, with sustainable lifestyle changes acting as a probable underlying mechanism.

## Figures and Tables

**Table 1:** Baseline characteristics of participants based on attendance vs. non-attendance at 6-, 12- and 24 months follow up.

	6 Months			12 Months			24 Months		
	Attendance	Non-Attendance	*p*-Value	Attendance	Non-Attendance	*p*-Value	Attendance	Non-Attendance	*p*-Value
n (%)	232 (68.6)	106 (31.4)		200 (59.2)	138 (40.8)		136 (40.2)	202 (59.8)	
Age, mean (SD)	52.0 (10.6)	52.5 (11.9)	0.75	52.3 (10.8)	51.9 (11.3)	0.75	53.5 (10.2)	51.3 (11.4)	0.07
Sex (female), n (%)	141 (60.8)	72 (67.9)	0.21	127 (63.5)	86 (62.3)	0.83	83 (61.0)	130 (64.4)	0.53
Type 2 Diabetes	115 (49.6)	53 (50.0)	0.94	98 (49.0)	70 (50.7)	0.76	65 (47.8)	103 (51.0)	0.56
Education level, n (%)									
None	37 (15.9)	18 (17.1)		34 (17.0)	21 (15.3)		28 (20.6)	27 (13.4)	
Short	57 (24.6)	27 (25.7)		52 (26.0)	32 (23.4)		33 (24.3)	51 (25.4)	
Medium	105 (45.3)	54 (51.4)		91 (45.5)	68 (49.6)		56 (41.2)	103 (51.2)	
Long	28 (12.1)	5 (4.8)		21 (10.5)	12 (8.8)		17 (12.5)	16 (8.0)	
Do not know	5 (2.2)	1 (1.0)	0.27	2 (1.0)	4 (2.9)	0.63	2 (1.5)	4 (2.0)	0.19
Married or registered partnership, n (%)	162 (69.8)	54 (51.4)	<0.001	141 (70.5)	75 (54.7)	<0.001	93 (68.4)	123 (61.2)	0.18
Employed or student, n (%)	171 (73.7)	69 (65.7)	0.13	147 (73.5)	93 (67.9)	0.26	100 (73.5)	140 (69.7)	0.44

**Table 2:** Change in weight from baseline to 6, 12 and 24 months in the intervention group compared to the control group in different subgroups.

	6 Months		12 Months		24 Months	
	Weight Change, kg [95% CI]		Weight Change, kg [95% CI]		Weight Change, kg [95% CI]	
	(Number)		(Number)		(Number)	
	Intervention	Control	Intervention	Control	Intervention	Control
All	3.99 [3.14; 4.84]	0.65 [−0.10; 1.39] **	4.57 [3.38; 5.76]	1.38 [0.13; 2.63] **	4.45 [2.79; 6.10]	2.54 [1.13; 3.95]
	(n = 148)	(n = 84)	(n = 127)	(n = 73)	(n = 81)	(n = 55)
Male	4.04 [2.56; 5.53]	0.57 [−0.57; 1.71] **	4.86 [2.75; 6.97]	1.38 [−0.27; 3.03] *	5.10 [1.79; 6.33]	2.58 [0.36; 4.66]
	(n = 52)	(n = 39)	(n = 41)	(n = 32)	(n = 30)	(n = 23)
Female	3.96 [2.90; 5.02]	0.71 [−0.30; 1.73] **	4.44 [2.97; 5.90]	1.37 [−9.50; 3.25] *	4.06 [2.75; 6.97]	2.51 [−0.27; 3.03]
	(n = 96)	(n = 45)	(n = 86)	(n = 41)	(n = 51)	(n = 32)
Age ≤ 54 years	3.86 [2.73; 4.98]	−0.18 [−1.21; 0.84] **	4.55 [2.79; 6.31]	1.04 [−0.70; 2.78] *	5.54 [2.83; 8.24]	1.46 [−0.66; 3.58] * ^e^
	(n = 87)	(n = 47)	(n = 73)	(n = 40)	(n = 43)	(n = 28)
Age > 54 years	4.18 [2.83; 5.53]	1.69 [0.67; 2.72] **	4.60 [3.07; 6.13]	1.78 [−0.10; 3.67] *	3.21 [1.41; 5.01]	3.66 [1.78; 5.55]
	(n = 61)	(n = 37)	(n = 54)	(n = 33)	(n = 38)	(n = 27)
BMI ≤ 34 kg/m^2^	3.81 [2.61; 5.02]	0.96 [−0.10; 2.02] **	4.09 [2.57; 5.60]	1.28 [−1.12; 3.68] *	3.57 [1.69; 5.45]	1.98 [−0.77; 4.72]
	(n = 69)	(n = 35)	(n = 61)	(n = 28)	(n = 42)	(n = 24)
BMI > 34 kg/m^2^	4.14 [2.92; 5.36]	0.42 [−0.63; 1.47] **	5.02 [3.18; 6.86]	1.44 [−0.02; 2.89] **	5.39 [2.55; 8.23]	2.98 [1.52; 4.44]
	(n = 79)	(n = 49)	(n = 66)	(n = 45)	(n = 39)	(n = 31)
T2D	4.00 [2.80; 5.20]	1.63 [0.58; 2.68] **	4.50 [3.04; 5.95]	2.09 [0.18; 4.00] *	5.35 [3.21; 7.50]	3.81 [1.92; 5.71]
	(n = 74)	(n = 41)	(n = 62)	(n = 36)	(n = 40)	(n = 25)
Without T2D	3.98 [2.74; 5.22]	−0.29 [−1.30; 0.72] **	4.64 [2.74; 6.55]	0.68 [−1.00; 2.37] **	3.56 [0.99; 6.13]	1.48 [−0.58; 3.53]
	(n = 74)	(n = 43)	(n = 65)	(n = 37)	(n = 41)	(n = 30)
Education level ^a^						
None	1.70 [−0.30; 3.70]	1.22 [−0.18; 2.62]	−3.34 [−0.40; 7.09]	0.99 [−1.19; 3.17]	5.28 [−1.15; 11.71]	0.66 [−1.89; 3.21]
	(n = 20)	(n = 17)	(n = 19)	(n = 15)	(n = 14)	(n = 14)
Short	5.99 [4.10; 7.88]	2.20 [−1.81; 1.81] ** ^b^	6.38 [3.88; 8.88]	1.37 [−1.64; 4.38] **	2.91 [0.04; 5.78]	3.48 [−1.43; 8.38]
	(n = 37)	(n = 20)	(n = 33)	(n = 19)	(n = 20)	(n = 13)
Medium	4.03 [2.84; 5.21]	0.56 [−0.70; 1.82] ** ^c^	4.32 [2.75; 5.89]	1.38 [−0.73; 3.49] *	4.98 [2.87; 7.09]	3.38 [1.90; 4.87]
	(n = 71)	(n = 34)	(n = 61)	(n = 30)	(n = 37)	(n = 19)
Long	2.88 [0.34; 5.42]	0.44 [−0.71; 1.59] **	3.95 [−0.69; 8.59]	2.02 [−1.99; 6.04]	5.06 [−1.86; 11.98]	2.33 [−1.25; 5.91]
	(n = 18)	(n = 10)	(n = 12)	(n = 9)	(n = 8)	(n = 9)
Marital status						
Married	4.15 [3.13; 5.18]	0.24 [−0.64; 1.12] **	4.90 [3.54; 6.25]	1.38 [−0.10; 2.86] **	3.77 [1.70; 5.84]	3.47 [1.70; 5.23] ^f^
	(n = 107)	(n = 55)	(n = 92)	(n = 49)	(n = 54)	(n = 39)
Single	3.57 [1.98; 5.15]	1.41 [0.03; 2.79] *	3.72 [1.20; 6.24]	1.37 [−1.11; 3.84]	5.80 [2.95; 8.64]	0.28 [−1.77; 2.33] **
	(n = 41)	(n = 29)	(n = 35)	(n = 24)	(n = 27)	(n = 16)
Employment						
Employed/student	3.83 [2.87; 4.79]	0.08 [−0.77; 0.94] **	4.59 [3.18; 6.00]	1.90 [0.34; 3.48] *	4.19 [2.53; 5.85]	2.78 [1.20; 4.36]
	(n = 113)	(n = 58)	(n = 97)	(n = 50)	(n = 61)	(n = 39)
Unemployed/retired	4.49 [2.58; 6.41]	1.90 [0.48; 3.32] *	4.51 [2.24; 6.78]	0.22 [−1.90; 2.34] **	5.22 [0.52; 9.93]	1.95 [−1.28; 5.18]
	(n = 35)	(n = 26)	(n = 30)	(n = 23)	(n = 20)	(n = 16)
Everyday physical activity						
<30 min	3.90 [2.08; 5.71]	−0.52 [−0.68; 1.71] **	5.60 [−8.54; −3.37]	0.01 [−2.12; 2.13] **	3.71 [−0.02; 7.43]	2.00 [−1.16; 5.10]
	(n = 30)	(n = 16)	(n = 25)	(n = 14)	(n = 19)	(n = 9)
30 min–2.5 h	3.82 [2.76; 4.89]	1.13 [0.04; 2.21] **	4.03 [−5.36; −2.70]	1.63 [−0.39; 3.64] *	4.49 [2.14; 6.82]	1.85 [−0.17; 3.87]
	(n = 78)	(n = 42)	(n = 67)	(n = 36)	(n = 41)	(n = 28)
>2.5 h	4.38 [2.30; 6.46]	0.58 [−0.97; 2.14] **	4.62 [1.50; 7.74]	1.82 [−0.47; 4.11]	5.04 [1.55; 8.53]	3.90 [1.08; 6.72]
	(n = 40)	(n = 26)	(n = 35)	(n = 23)	(n = 21)	(n = 18)
Physical exercise						
<30 min	4.20 [3.20; 5.20]	0.05 [−0.89; 1.00] **	5.07 [3.56; 6.58]	0.62 [−0.82; 2.05] **	4.11 [1.80; 6.42]	2.00 [0.24; 3.77]
	(n = 93)	(n = 51)	(n = 78)	(n = 44)	(n = 48)	(n = 33)
30 min–2 h	3.24 [1.68; 4.80]	1.62 [−3.03; −0.22] ^d^	3.64 [2.13; 5.15]	2.13 [−0.59; 4.84]	4.42 [1.77; 7.07]	3.41 [0.44; 6.37]
	(n = 42)	(n = 27)	(n = 38)	(n = 23)	(n = 24)	(n = 18)
>2 h	4.91 [−0.05; 9.87]	1.28 [−1.52; 4.09]	4.25 [−3.96; 12.46]	4.08 [−1.82; 9.98]	6.31 [−0.05; −12.67]	3.08 [−3.00; 9.15]
	(n = 13)	(n = 6)	(n = 11)	(n = 6)	(n = 9)	(n = 4)
Diet						
Unhealthy	3.96 [2.87; 5.05]	0.71 [−0.17; 1.60] **	4.93 [3.34; 6.52]	1.59 [0.26; 2.92] **	4.20 [1.77; 6.63]	2.10 [0.63; 3.57]
	(n = 84)	(n = 47)	(n = 72)	(n = 41)	(n = 47)	(n = 33)
Healthy	4.03 [2.64; 5.42]	0.56 [−0.75; 1.87] **	4.10 [2.26; 5.94]	1.10 [−1.28; 3.48] *	4.79 [2.58; 6.99]	3.20 [0.30; 6.09]
	(n = 64)	(n = 37)	(n = 55)	(n = 32)	(n = 34)	(n = 22)

Abbreviations: BMI; body mass index, T2D; type 2 diabetes; *p*-values comparing weight loss between the intervention and the control group (* *p* < 0.05 and ** *p* < 0.01); ^a^ Three participants in the control group and two participants in the intervention group did not know their education level; ^b^ *p* = 0.006 for the interaction between short education and weight loss in the intervention group vs. control group at 6 months; ^c^ *p* = 0.10 for the interaction between medium education and weight loss in intervention group vs. control group at 6 months; ^d^ *p* = 0.08 for the interaction between physical exercise and weight loss in the intervention group vs. control group at 6 months; ^e^ *p* = 0.052 for the interaction between age and weight loss in the intervention group vs. control group at 24 month; ^f^ *p* = 0.04 for the interaction between marital status and weight loss in the intervention group vs. control group at 24 month.

**Table 3:** Group comparison of number of participants reporting improvements from baseline in physical and dietary behaviors at 6-, 12- and 24 months follow-up stratified by education level.

	Baseline to 6 Months		Baseline to 12 Months		Baseline to 24 Months	
	Improved/Total, (%)		Improved/Total, (%)		Improved/Total, (%)	
	Intervention	Control	Intervention	Control	Intervention	Control
Physical exercise						
No education	10/22 (45.5)	8/17 (47.1)	11/18 (61.1)	8/15 (53.3)	7/14 (50.0)	5/11 (45.5)
Short education	23/38 (60.5)	13/22 (59.1)	18/31 (58.1)	10/19 (52.6)	14/24 (58.3)	7/13 (53.9)
Medium education	36/74 (48.7)	17/42 (40.5)	26/58 (44.8)	13/31 (41.9)	18/41 (43.9)	8/20 (40.0)
Long education	6/17 (35.3)	3/11 (27.3)	9/13 (69.2)	2/9 (22.2) *	3/9 (33.3)	0/7 (0.0)
Everyday physical activity						
No education	11/22 (50)	11/17 (64.7)	10/18 (55.6)	9/15 (60.0)	5/14 (35.7)	6/11 (54.6)
Short education	19/38 (50)	5/22 (22.7) *	14/31 (45.2)	6/19 (31.6)	13/24 (54.2)	7/13 (53.9)
Medium education	34/74 (46.0)	12/42 (28.6)	30/58 (51.7)	9/31 (29.0) *	18/41 (43.9)	8/20 (40.0)
Long education	6/17 (35.3)	7/11 (63.6)	5/13 (38.5)	4/9 (44.4)	5/9 (55.6)	2/7 (28.6)
Dietary habits—Vegetables and/or root vegetables						
No education	8/22 (36.36)	5/17 (29.4)	10/18 (55.6)	5/15 (33.3)	3/14 (21.4)	4/11 (36.4)
Short education	19/38 (50)	5/22 (22.7) *	15/31 (48.4)	7/19 (36.8)	9/24 (37.5)	4/13 (30.8)
Medium education	36/74 (48.65)	10/42 (23.8) **	22/58 (37.9)	11/31 (35.5)	21/41 (51.2)	10/20 (50.0)
Long education	2/17 (11.76)	3/11 (27.3)	4/13 (30.8)	1/9 (11.1)	0/9 (0.0)	3/7 (42.9) *
Dietary habits—Fruit and/or berries						
No education	7/22 (31.8)	4/17 (23.5)	8/18 (44.4)	6/15 (40.0)	6/14 (42.9)	5/11 (44.0)
Short education	14/38 (36.8)	6/22 (27.3)	14/31 (45.2)	5/19 (26.3)	8/24 (33.3)	1/13 (7.7)
Medium education	28/74 (37.8)	9/42 (21.4)	16/58 (27.6)	9/31 (29.0)	13/41 (31.7)	7/20 (35.0)
Long education	2/17 (11.8)	2/11 (18.2)	4/13 (30.8)	2/9 (22.2)	2/9 (22.2)	3/7 (42.9)
Dietary habits—Seafood						
No education	4/22 (18.2)	5/17 (29.4)	6/18 (33.3)	5/15 (33.3)	3/14 (21.4)	5/11 (45.5)
Short education	11/38 (29.0)	5/22 (22.7)	11/31 (35.5)	4/19 (21.1)	7/24 (28.2)	4/13 (30.8)
Medium education	23/74 (31.1)	9/42 (21.4)	16/58 (27.6)	9/31 (29.0)	6/41 (14.6)	6/20 (30.0)
Long education	4/17 (23.5)	3/11 (27.3)	5/13 (38.5)	3/9 (33.3)	3/9 (33.3)	4/7 (57.1)
Dietary habits—Pastry, chocolate, sweets, soda						
No education	8/22 (36.4)	10/17 (58.8)	10/18 (55.6)	5/15 (33.3)	4/14 (28.6)	5/11 (45.5)
Short education	23/38 (60.5)	11/22 (50.0)	13/31 (41.9)	6/19 (31.6)	7/24 (29.2)	5/14 (35.7)
Medium education	39/74 (52.7)	11/42 (26.2) **	30/58 (51.7)	14/31 (45.2)	17/42 (40.5)	7/21 (33.3)
Long education	3/17 (17.7)	5/11 (45.5)	5/13 (38.5)	3/9 (33.3)	0/12 (0.0)	5/8 (62.5) **

*p*-values comparing the fraction of participants improving lifestyle behaviors between the intervention and the control group (* *p* < 0.05 and ** *p* < 0.01).

**Table 4:** Associations between weight loss and baseline characteristics and changes in physical and dietary behaviors between baseline and 6 months.

	All Participants	Intervention	Control
	Beta-Coefficient [95% CI]	Beta-Coefficient [95% CI]	Beta-Coefficient [95% CI]
Age (years)	0.07 [0.01; 0.13] *	0.076 [−0.01; 0.16]	0.041 [−0.020; 0.10]
Sex (male vs. female)	−0.37 [−1.68; 0.94]	0.085 [−1.71; 1.88]	−0.15 [−1.64; 1.35]
BMI (kg/m^2^)	−0.18 [−0.35; −0.01] *	−0.10 [−0.33; 0.13]	−0.21 [−0.41; −0.010] *
Without T2D vs. with T2D	−0.75 [−2.02; 0.53]	−0.02 [−1.73; 1.69]	−1.91 [−3.35; −0.48] **
Education level			
None vs. medium	−1.43 [−3.27; 0.42]	−2.33 [−4.88; 0.22]	0.66 [−1.38; 2.69]
Short vs. medium	0.98 [−0.61; 2.57]	1.96 [−0.08; 4.00]	−0.56 [−2.49; 1.37]
Long vs. medium	−0.89 [−2.95; 1.16]	−1.14 [- 3.80; 1.51]	−0.12 [−2.58; 2.35]
Married or registered partnership vs. single, divorced or widow	0.15 [−1.24; 1.54]	0.59 [−1.32; 2.49]	−1.17 [−2.72; 0.38]
Employed or student vs. Unemployed or retired	−0.82 [−2.27; 0.63]	−0.66 [−2.68; 1.35]	−1.81 [−3.38; −0.24] *
Physical exercise			
Stable vs. worsened	1.65 [−0.39; 3.68]	2.70 [−0.03; 5.42] *	−0.09 [−2.42–2.24]
Improved vs. worsened	1.70 [−0.28; 3.67]	2.11 [−0.52; 4.74]	0.76 [−1.52–3.05]
Everyday physical activity			
Stable vs. worsened	1.93 [0.24; 3.62] *	1.64 [−0.66; 3.94]	1.41 [−0.57; 3.40]
Improved vs. worsened	2.35 [0.81; 3.88] **	2.77 [0.65; 4.90] *	0.44 [−1.30; 2.18]
Dietary habits—Vegetables and/or root vegetables			
Stable vs. worsened	0.82 [−1.26; 2.90]	1.49 [−1.57; 4.55]	−0.39 [−2.57; 1.79]
Improved vs. worsened	3.20 [1.06; 5.34] **	4.09 [1.01; 7.17] *	0.057 [−2.34; 2.46]
Dietary habits—Fruit and/or berries			
Stable vs. worsened	1.22 [−0.60; 3.05]	0.98 [−1.66; 3.63]	0.79 [−1.12; 2.69]
Improved vs. worsened	2.08 [0.10; 4.06] *	1.24 [−1.53; 4.02]	1.73 [−0.54; 4.01]
Dietary habits—Seafood			
Stable vs. worsened	−0.79 [−3.71; 2.13]	−0.10 [−4.18; 3.98]	−2.21 [−5.30; 0.89]
Improved vs. worsened	1.15 [−1.94; 4.23]	1.52 [−2.76; 5.80]	0.04 [−3.27; 3.34]
Dietary habits—Pastry, chocolate, sweets, soda			
Stable vs. worsened	0.90 [−1.24; 3.05]	2.81 [0.095; 5.53] *	−1.94 [−4.70; 0.83]
Improved vs. worsened	2.31 [0.16; 4.45] *	3.68 [1.02; 6.35] **	−0.58 [−3.34; 2.23]
Intervention vs. control	3.34 [2.09; 4.60] **		

*p*-values for linear associations between weight loss and baseline characteristics and changes in physical and dietary behaviors: * *p* < 0.05 and ** *p* < 0.01; Abbreviations: BMI; body mass index, T2D; type 2 diabetes.

## Data Availability

The data presented in this study are available on request from the corresponding author. The data are not publicly available due to restrictions on privacy.

## Supplementary Materials

The following supporting information can be downloaded at: https://www.mdpi.com/article/10.3390/nu16060795/s1, Table S1: Patient questionnaire on sociodemographic data and lifestyle habits at baseline and at 6, 12 and 24 months. Table S2: Associations between weight loss and baseline characteristics and changes in physical and dietary behaviors between baseline and 6 months using multiple regression analyses. Table S3: Associations between weight loss and baseline characteristics and changes in physical and dietary behaviors between baseline and twelve months using multiple regression analyses. Table S4: Associations between weight loss and baseline characteristics and changes in physical and dietary behaviors between baseline and twelve months. Table S5: Associations between weight loss and baseline characteristics and changes in physical and dietary behaviors between baseline and twenty-four months.

## References

[1] Beckman J.A., Creager M.A. (2016). Vascular Complications of Diabetes. Circ. Res..

[2] Videncenter for Diabetes Diabetes i tal. https://videncenterfordiabetes.dk/viden-om-diabetes/generelt-om-diabetes/diabetes-i-tal.

[3] Kristensen J.K. Type 2-Diabetes: Opfølgning og Behandling. https://www.dsam.dk/vejledninger/type2.

[4] Knowler W.C., Barrett-Connor E., Fowler S.E., Hamman R.F., Lachin J.M., Walker E.A., Nathan D.M. (2002). Reduction in the incidence of type 2 diabetes with lifestyle intervention or metformin. N. Engl. J. Med..

[5] Bo A., Thomsen R.W., Nielsen J.S., Nicolaisen S.K., Beck-Nielsen H., Rungby J., Sørensen H.T., Hansen T.K., Søndergaard J., Friborg S. (2018). Early-onset type 2 diabetes: Age gradient in clinical and behavioural risk factors in 5115 persons with newly diagnosed type 2 diabetes-Results from the DD2 study. Diabetes Metab. Res. Rev..

[6] du Pon E., Wildeboer A.T., van Dooren A.A., Bilo H.J.G., Kleefstra N., van Dulmen S. (2019). Active participation of patients with type 2 diabetes in consultations with their primary care practice nurses—What helps and what hinders: A qualitative study. BMC Health Serv. Res..

[7] Hutchesson M.J., Rollo M.E., Krukowski R., Ells L., Harvey J., Morgan P.J., Callister R., Plotnikoff R., Collins C.E. (2015). eHealth interventions for the prevention and treatment of overweight and obesity in adults: A systematic review with meta-analysis. Obes. Rev..

[8] Sherrington A., Newham J.J., Bell R., Adamson A., McColl E., Araujo-Soares V. (2016). Systematic review and meta-analysis of internet-delivered interventions providing personalized feedback for weight loss in overweight and obese adults. Obes. Rev..

[9] Juul L. Overvægt og Fedme. https://www.sundhed.dk/borger/patienthaandbogen/hormoner-og-stofskifte/sygdomme/overvaegt-og-kost/overvaegt-og-fedme/#:~:text=Verdenssundhedsorganisationen%20WHO%20har%20defineret%20gr%C3%A6nser%20for%20normalv%C3%A6gt%20og,6%20BMI%20%E2%89%A540%20define.

[10] Hesseldal L., Christensen J.R., Olesen T.B., Olsen M.H., Jakobsen P.R., Laursen D.H., Lauridsen J.T., Nielsen J.B., Søndergaard J., Brandt C.J. (2022). Long-term Weight Loss in a Primary Care-Anchored eHealth Lifestyle Coaching Program: Randomized Controlled Trial. J. Med. Internet Res..

[11] Donkin L., Christensen H., Naismith S.L., Neal B., Hickie I.B., Glozier N. (2011). A systematic review of the impact of adherence on the effectiveness of e-therapies. J. Med. Internet Res..

[12] Myers-Ingram R., Sampford J., Milton-Cole R., Jones G.D. (2023). Effectiveness of eHealth weight management interventions in overweight and obese adults from low socioeconomic groups: A systematic review. Syst. Rev..

[13] Brandt C.J., Christensen J.R., Lauridsen J.T., Nielsen J.B., Søndergaard J., Sortsø C. (2020). Evaluation of the Clinical and Economic Effects of a Primary Care Anchored, Collaborative, Electronic Health Lifestyle Coaching Program in Denmark: Protocol for a Two-Year Randomized Controlled Trial. JMIR Res. Protoc..

[14] Haste A., Adamson A.J., McColl E., Araujo-Soares V., Bell R. (2017). Web-Based Weight Loss Intervention for Men With Type 2 Diabetes: Pilot Randomized Controlled Trial. JMIR Diabetes.

[15] Fredriksson E., Brekke H.K., Ellegård L. (2014). Validation of four questions on food habits from the Swedish board of health and social welfare by 3-day food records in medical and nursing students. Food Nutr. Res..

[16] Ryan P. (2009). Integrated Theory of Health Behavior Change: Background and intervention development. Clin. Nurse Spec..

[17] Prochaska J.O., Velicer W.F. (1997). The transtheoretical model of health behavior change. Am. J. Health Promot..

[18] Brandt C.J., Clemensen J., Nielsen J.B., Søndergaard J. (2018). Drivers for successful long-term lifestyle change, the role of e-health: A qualitative interview study. BMJ Open.

[19] Bandura A. (2004). Health promotion by social cognitive means. Health Educ. Behav..

[20] Ryan R.M., Deci E.L., Maggino F. (2020). Self-Determination Theory. Encyclopedia of Quality of Life and Well-Being Research.

[21] Epstein R.M., Franks P., Fiscella K., Shields C.G., Meldrum S.C., Kravitz R.L., Duberstein P.R. (2005). Measuring patient-centered communication in patient-physician consultations: Theoretical and practical issues. Soc. Sci. Med..

[22] Michie S., Richardson M., Johnston M., Abraham C., Francis J., Hardeman W., Eccles M.P., Cane J., Wood C.E. (2013). The behavior change technique taxonomy (v1) of 93 hierarchically clustered techniques: Building an international consensus for the reporting of behavior change interventions. Ann. Behav. Med..

[23] Miller W.R., Rose G.S. (2009). Toward a theory of motivational interviewing. Am. Psychol..

[24] Coupe N., Cotterill S., Peters S. (2018). Tailoring lifestyle interventions to low socio-economic populations: A qualitative study. BMC Public. Health.

[25] Bennett G.G., Warner E.T., Glasgow R.E., Askew S., Goldman J., Ritzwoller D.P., Emmons K.M., Rosner B.A., Colditz G.A. (2012). Obesity treatment for socioeconomically disadvantaged patients in primary care practice. Arch. Intern. Med..

[26] Serdula M.K., Mokdad A.H., Williamson D.F., Galuska D.A., Mendlein J.M., Heath G.W. (1999). Prevalence of attempting weight loss and strategies for controlling weight. JAMA.

[27] Earnest C.P., Church T.S. (2020). A Retrospective Analysis of Employee Education Level on Weight Loss Following Participation in an Online, Corporately Sponsored, Weight Loss Program. J. Occup. Environ. Med..

