# Supplementary material for: The Impact of Education Level on Weight Loss in a Primary Care-Anchored eHealth Lifestyle Coaching Program in Denmark: A Randomized Controlled Trial
Source: Nutrients. 2024 Mar 11;16(6):795. doi: 10.3390/nu16060795 (PMC10975873; doi:10.3390/nu16060795)
Supplement: Supplementary file 1 [file nutrients-16-00795-s001.zip › nutrients-2824516-supplementary.pdf]

Table S1. Patient questionnaire on sociodemographic data and lifestyle habits at baseline and at 6,12 and 24 months.

**1. Select below your highest completed level of education**	
None = No higher education	
Short = Short higher education of 2 years (ex. business academy)	
Medium = Medium-term higher education of 3-4.5 years (ex. professional bachelor)	
Long = Long higher education of at least 5 years (ex. graduate and Ph.D. at the university)	

**2. Select your marital status**	
Married or registered partnership	
Single	
Widow or longest living partner	
Divorce or no longer in registered partnership	

**3. Select your employment status**	
On the labor market (employed or self-employed)	
Unemployed (maternity leave or on unemployment benefits)	
Unemployed (cash benefits)	
Student	
Early retirement pension	
Retired	

**4. How often do you eat vegetables and / or root vegetables (fresh as well as frozen)?**	
Twice daily or more often	
Once a day	
Sometimes during the week	
Once a week or less frequently	

**5. How often do you eat fruit and / or berries (fresh, frozen, preserves, or juice / smoothie)?**	
Twice daily or more often	
Once a day	
Sometimes during the week	
Once a week or less frequently	

**6. How often do you eat seafood as a main course?**	
Three times a week or more often	
Twice a week	
Once a week	
A few times a month or less	

**7. How often do you eat pastry, chocolate, sweets and / or drink soda?**	
Daily	
Almost every day	
A couple of times a week	
Once a week or less frequently	

**8. How much time do you spend per week on physical exercise that makes you breathless? (For example, running, football, aerobics, tennis, jogging, or the like)**	
0 minutes / no time	
Less than 30 minutes	
30-60 minutes (½-1 hour)	
60-120 minutes (1-2 hours)	
More than 120 minutes (2 hours or more)	

**9. How much time do you spend per week on everyday physical activity? (For example, a walk, light gardening, cleaning, cycling, to and from work or the like)**	
0 minutes / No time	
Less than 30 minutes	
30-60 minutes (½ - 1 hour)	
60-90 minutes (1 - 1½ hour)	
90-150 minutes (1½ - 2½ hours)	
150-300 minutes (2½ - 5 hours)	
More than 300 minutes (5 hours or more)	

Table S2. Associations between weight loss and baseline characteristics and changes in physical and dietary behaviors between baseline and six months using multiple regression analyses.

	Multivariat regression					
	All participants		Intervention		Control	
	**Beta koefficient**	**P-value**	**Beta koefficient**	**P-value**	**Beta koefficient**	**P-value**
**Age (years)**	0.06<br>[0.0065 to 0.12]	0.029				
**Education level**						
None vs. medium			-1.82<br>[-4.32 - 0.67]	0.15		
Short vs. medium			1.81<br>[-0.19 - 3.81]	0.076		
Long vs. medium			0.46<br>[-2.32 - 3.24]	0.74		
**Everyday physical activity**						
Stable vs. worsen	1.57<br>[-0.0018 - 3.15]	0.05	1.92<br>[-0.32 - 4.16]	0.092		
Improved vs. worsen	1.68<br>[0.24 - 3.11]	0.022	2.60<br>[0.54 - 4.66]	0.014		
**Dietary habits – Vegetables and / or root vegetables**						
Stable vs. worsen	0.75<br>[-1.20 - 2.70]	0.45	1.21<br>['-1.75 - 4.18]	0.42		
Improved vs. worsen	2.46<br>[0.43 - 4.49]	0.018	3.84<br>[0.83 - 6.84]	0.013		
**Dietary habits - Pastry, chocolate, sweets, soda**						
Stable vs. worsen	0.98<br>[-1.02 - 2.97]	0.34	2.24<br>[-0.38 - 4.88]	0.093		
Improved vs. worsen	1.92<br>[-0.057 - 3.90]	0.057	2.39<br>[-0.27 - 5.06]	0.078		

**Randomization, intervention**	2.94<br>[1.66 - 4.21]	<0.001				
**Obesity vs. Diabetes**					-1.92<br>[-3.35 - -0.48]	0.009
**Adjusted R-square**	0.19		0.16		0.068	

Table S3. Associations between weight loss and baseline characteristics and changes in physical and dietary behaviors between baseline and twelve months using multiple regression analyses.

	12 months Multivariat regression					
	All participants		Intervention		Control	
	Beta koefficient	P-value	Beta koefficient	P-value	Beta koefficient	P-value
**Age (years)**	0.08 [-0.001 - 0.16]	0.05				
**Everyday activity**						
Stable vs. worsen	2.66 [0.35 - 4.97]	0.02	3.72 [0.48 - 6.97]	0.03		
Improved vs. worsen	2.49 [0.47 - 4.50]	0.02	2.67 [-0.10 - 5.44]	0.059		
**Dietary habits - Pastry, chocolate, sweets, soda**						
Stable vs. worsen	2.44 [-0.65 - 5.54]	0.12			2.27 [-1.55 - 6.08]	0.24
Improved vs. worsen	4.82 [1.67 - 7.97]	0.003			5.70 [1.69 - 9.72]	0.006
**Randomization, intervention**	2.75 [0.97 - 4.54]	0.003				
**Adjusted R-square**	0.13		0.03		0.12	

Table S4. Associations between weight loss and baseline characteristics and changes in physical and dietary behaviors between baseline and twelve months.

	12 months Univariat regression					
	All participants		Intervention		Control	
	Beta koefficient [95% CI]	P-value	Beta koefficient [95% CI]	P-value	Beta koefficient [95% CI]	P-value
**Age (years)**	0.07 [-0.01 - 0.15]	0.10	0.09 [-0.03 - 0.21]	0.13	0.04 [-0.060 - 0.15]	0.40
**Sex (male vs. female)**	-0.11 [-1.99 - 1.76]	0.90	0.42 [-2.13 - 2.97]	0.75	0.01 [-2.53 - 2.55]	0.10
**BMI (kg/m^2)**	-0.17 [-0.41 - 0.07]	0.15	-0.11 [-0.44 - 0.21]	0.48	-0.12 [-0.45 - 0.22]	0.49
**Without T2D vs. with T2D**	-0.41 [-2.21 - 1.40]	0.66	0.15 [-2.24 – 2.53]	0.90	-1.41 [-3.90 - 1.09]	0.27
**Education level**						
None vs. medium	-1.05 [-3.61 - 1.52]	0.42	-0.98 [-4.48 - 2.53]	0.58	-0.39 [-3.83 - 3.06]	0.82
Short vs. medium	1.20 [-1.02 - 3.41]	0.29	2.06 [-0.83 - 4.94]	0.16	-0.01 [-3.21 - 3.18]	0.99
Long vs. medium	0.23 [-3.31 - 2.86]	0.88	-0.37 [-4.58 - 3.84]	0.86	0.64 [-3.50 - 4.79]	0.76
**Married or registered partnership vs. single, divorced or widow**	0.9 [-1.07 - 2.89]	0.36	1.18 [-1.49 - 3.84]	0.38	0.02 [-2.67 - 2.70]	0.99
**Employed or student vs. unemployed or retired**	1.03 [-1.01 - 3.07]	0.32	0.08 [-2.73 - 2.89]	0.96	1.69 [-0.99 - 4.37]	0.21
**Physical exercise**						
Stable vs. worsen	-0.52	0.73	-0.70	0.03	-0.61	0.76

	[-3.51 - 2.47]		[-4.83 - 3.43]		[-4.49 - 3.28]	
Improved vs. worsen	0.30	0.84	0.26	0.74	-0.63	0.75
	[-2.61 - 3.21]		[-3.74 - 4.26]		[-4.48 - 3.23]	
**Everyday physical activity**						
Stable vs. worsen	2.19	0.08	3.72	0.90	-0.15	0.93
	[-0.24 - 4.61]		[0.48 - 6.97]		[-3.42 - 3.13]	
Improved vs. worsen	2.37	0.03	2.67	0.059	1.34	0.38
	[0.24 - 4.49]		[-0.10 - 5.44]		[-1.67 - 4.34]	
**Dietary habits – Vegetables and / or root vegetables**						
Stable vs. worsen	0.96	0.54	0.89	0.71	0.33	0.86
	[-2.16 - 4.08]		[-3.77 - 5.55]		[-3.44 - 4.09]	
Improved vs. worsen	2.42	0.14	2.55	0.28	0.34	0.87
	[-0.77 - 5.61]		[-2.14 - 7.24]		[-3.71 - 4.40]	
**Dietary habits – Fruit and / or berries**						
Stable vs. worsen	0.15	0.90	-0.30	0.86	0.06	0.97
	[-2.23 - 2.54]		[-3.60 - 3.00]		[-3.07 - 3.19]	
Improved vs. worsen	1.97	0.13	2.01	0.25	0.47	0.79
	[-0.57 - 4.51]		[-1.45 - 5.48]		[-3.00 - 3.94]	
**Dietary habits – Seafood**						
Stable vs. worsen	-1.04	0.61	0.31	0.89	-1.91	0.70
	[-5.03 - 2.94]		[-4.25 - 4.87]		[-11.68 - 7.86]	
Improved vs. worsen	2.47	0.25	2.85	0.24	2.79	0.58
	[-1.70 - 6.63]		[-1.95 - 7.66]		[-7.14 - 12.73]	
**Dietary habits - Pastry, chocolate, sweets, soda**						
Stable vs. worsen	2.44	0.14	2.15	0.37	2.27	0.24
	[-0.77 - 5.65]		[-2.53 - 6.82]		[-1.55 - 6.08]	
Improved vs. worsen	4.85	0.004	3.51	0.14	5.70	0.006
	[1.61 - 8.09]		[-1.15 - 8.16]		[1.69 - 9.72]	
**Intervention vs. control**	3.19	0.001				
	[1.37 - 5.02]					

Table S5. Associations between weight loss and baseline characteristics and changes in physical and dietary behaviors between baseline and twenty-four months.

	24 months Multivariat regression					
	**All participants**		**Intervention**		**Control**	
	**Beta koefficient**	**P-value**	**Beta koefficient**	**P-value**	**Beta koefficient**	**P-value**
**Age (years)**					0.13 [0.01 - 0.25]	0.03
**Dietary habits - Pastry, chocolate, sweets, soda**						
Stable vs. worsen	4.95 [0.80 - 9.10]	0.02	5.61 [-0.12 - 11.35]	0.055		
Improved vs. worsen	4.56 [0.19 - 8.94]	0.04	6.06 [-0.24 - 12.37]	0.059		
**Adjusted R-square**	0.05		0.03		0.07	
